# Join the club: YIPF3 and YIPF4 act as Golgiphagy receptors

**DOI:** 10.1093/lifemeta/load049

**Published:** 2023-12-09

**Authors:** Xiaoli Ma, Hong Zhang

**Affiliations:** National Laboratory of Biomacromolecules, New Cornerstone Science Laboratory, Institute of Biophysics, Chinese Academy of Sciences, Beijing 100101, China; National Laboratory of Biomacromolecules, New Cornerstone Science Laboratory, Institute of Biophysics, Chinese Academy of Sciences, Beijing 100101, China; College of Life Sciences, University of Chinese Academy of Sciences, Beijing 100049, China

**Autophagic degradation of membrane-bound organelles such as the endoplasmic reticulum (ER), mitochondria, and peroxisomes is mediated by a family of receptors. In a recent study, Hickey *et al.* revealed that autophagy triggered by nutrient stress, previously thought to degrade bulk cytoplasmic contents, prioritizes the ER and Golgi as cargoes for degradation. They further demonstrated that Yip1 domain family member 3 (YIPF3) and YIPF4 act as receptors to mediate the delivery of Golgi to the lysosomes for Golgi remodelling during nutrient stress and also neuronal differentiation.**

Macroautophagy, hereafter referred to as autophagy, involves engulfment of cytosolic constituents within a *de novo* synthesized double-membrane autophagosome, which is subsequently transported to the lysosome for degradation and recycling of sequestered materials [1, 2]. Autophagosomes can capture specific cargoes such as protein aggregates and superfluous/damaged organelles to maintain cellular homeostasis [3]. According to the type of cargoes sequestrated into autophagosomes, selective autophagy can be categorized into aggrephagy (protein aggregates as cargoes), ERphagy (ER as cargoes), mitophagy (mitochondria as cargoes), and lysophagy (lysosomes as cargoes) [3]. A family of receptor proteins has been identified that mediate the engagement of specific cargoes with the isolation membrane (IM, autophagosomal precursor) [4]. The receptors recognize cargoes directly or indirectly such as via binding to specific tags (e.g. polyubiquitin chains) conjugated to the cargoes [3]. The receptors also bind to the microtubule-associated protein 1 light chain 3 (LC3)/γ-aminobutyric acid type A (GABA_A_) receptor-associated protein (GABARAP) family of autophagy proteins, which are orthologs of the archetypal yeast autophagy protein autophagy-related gene (ATG) 8. The LC3/GABARAP proteins are conjugated to phosphatidylethanolamine (PE) on the autophagosome precursor membrane, called IM [5]. The receptor proteins can also recruit upstream-acting autophagy proteins such as focal adhesion kinase family interacting protein of 200 kDa (FIP200) to trigger the formation of surrounding autophagic structures [6]. Different receptors are utilized for degradation of diverse cargoes. For example, p62 (also called sequestosome 1 (SQSTM1)), NBR1 (neighbour of breast cancer gene 1 (*BRCA1*) gene 1), TAX1BP1 (TAX1-binding protein-1, also called tumour necrosis factor receptor associated factor 6-binding protein (T6BP)), and SEPA-1 (suppressor of ectopic P granule in autophagy mutants 1) facilitate the degradation of various protein cargoes [3]. FUNDC1 (FUN14 domain containing 1), CALCOCO2/NDP52 (calcium binding and coiled-coil domain-containing protein 2/nuclear dot 10 protein 52), and OPTN (optineurin) act as receptors for mitophagy [3], while FAM134B (family with sequence similarity 134, member B, also called reticulophagy regulator 1 (RETREG1)), RTN3 (reticulon 3), CCPG1 (cell-cycle progression gene 1), SEC62, TEX264 (testes expressed gene 264), and ATL3 (atlastin GTPase 3) mediate ERphagy [6]. The Golgi complex serves as a hub for reception and dispatch of newly synthesized proteins from the ER via vesicle-mediated transport. ER luminal or ER transmembrane proteins are encapsulated within coat protein complex II (COPII) vesicles and subsequently transported to the Golgi apparatus. Following fusion with the Golgi, they are post-translationally modified, sorted, and directed to distinct destinations. The Golgi apparatus is also involved in lipid transport and lysosome formation. Whether and how the Golgi complex is selectively degraded by autophagy remains largely unknown.

Autophagy plays a pivotal function in cell survival during nutrient stress. Starvation-induced autophagy is generally regarded as a non-selective process, in which a portion of the cytosol is indiscriminately engulfed for degradation. This prevailing hypothesis is challenged by the observation that ERphagy is elevated by starvation. Very little is known about the extent of selectivity during cargo sequestration by the autophagosome. For example, is the fraction of protein molecules degraded by autophagy correlated with their total abundance within the cell and across individual subcellular compartments? Is the Golgi complex, whose functions in protein modification and secretion appear to be less in demand under starvation, prioritized for degradation? If yes, do any Golgi-localized transmembrane proteins act as receptors, analogous to ERphagy and mitophagy?

A study of Hickey and colleagues systemically investigated specific proteins, protein complexes, and organelles that are susceptible to autophagic degradation upon nutrient stress [7]. The researchers performed a comprehensive proteome census of nutrient stress-triggered autophagy. Proteome analysis was conducted in control and autophagy-deficient cell lines (*FIP200*^*–/–*^ or *ATG7*^*–/–*^) under nutrient depletion conditions (Earle’s balanced salt solution (EBSS) treatment or amino acid starvation). Out of approximately 8000 quantified proteins, 732 and 684 were identified as candidate autophagy proteins (CAPs), exhibiting decreased abundance profiles with EBSS treatment or amino acid withdrawal, respectively. When the CAPs were subjected to gene ontology (GO) analysis, the top 10 GO terms were enriched in ER- and Golgi-related functions. Comparison of the two distinct starvation conditions identified 187 overlapping CAPs, which were particularly enriched in Golgi- and ER-localized proteins. The authors further investigated how the number of CAP molecules degraded by autophagy is related to total protein abundance. When considering the absolute abundance of CAPs, they found that the majority of copies degraded are contributed by the cytosol, ER, endosomes, and Golgi. During nutrient deprivation, the cellular proteome is also depleted by non-autophagy survival mechanisms, including proteasomal degradation and translation inhibition. Therefore, for each subcellular compartment in nutrient-deprived cells, the authors calculated the percentage of CAPs out of the total number of lost proteins. Surprisingly, only about 3% of lost cytosolic proteins are CAPs, compared to ~27% for the ER and ~79% for the Golgi membrane [7]. Although the ER and Golgi harbour only approximately 5.2% of protein copies per cell, these organelles account for around 50% of all CAP copies lost. The comprehensive proteome census conducted in this study argues that nutrient stress triggers autophagy of specific organelles, not non-selective bulk autophagic degradation.

The authors then investigated whether Golgiphagy is mediated by membrane-embedded receptors. The receptors involved in organelle autophagic turnover bind to the targeted organelles and autophagy proteins. The receptors are also transported to the lysosomes with the cargo. Proximity biotinylation of engineered ascorbate peroxidase 2 (APEX2)-LC3B and APEX2-GABARAPL2 (GABARAP-like 2) was employed to identify Golgi proteins that show LC3-interaction region (LIR)-dependent proximity to ATG8-related proteins [7]. Combining the data from proximity labelling with the extent of starvation- and autophagy-dependent degradation, the authors identified the Golgi-resident protein Yip1 domain family member 4 (YIPF4). For follow-up studies, they also included YIPF3, which exhibits a similar abundance profile to other well-characterized receptors in global proteomics experiments. YIPF3 and YIPF4 are Golgi-localized transmembrane proteins with a cytosolic amino-terminal region harbouring a potential LIR (Fig. 1). AlphaFold predicts that YIPF3 forms a heterodimer with YIPF4. Heterodimer formation is required for YIPF3 stability. Proximity biotin labelling of APEX2-YIPF3 and APEX2-YIPF4 also revealed the enrichment of GABARAPL1 (GABARAP-like 1), WIPI1/2 (WD-repeat protein interacting with phosphoinositides 1/2), ATG3, and ATG4B among YIPF3/4-interacting proteins during nutrient stress. The interaction of YIPF3 and YIPF4 with GABARAPL1 was LIR-dependent. Fusion of YIPF3 and YIPF4 to the pH-sensitive fluorophore Keima revealed trafficking to the acidic lysosomes in a manner dependent on FIP200, which is essential for autophagosome formation. Furthermore, degradation of YIPF3/4 proteins in response to nutrient stress requires GABARAP but not LC3. The monomeric neon green fluorescent protein (mNEON)-YIPF4 reporter showed colocalization with the Golgi marker golgin subfamily B member 1 (GOLGB1) and did not exhibit a preference for *cis-* or *trans-*Golgi. Following 3-h starvation, mNEON-YIPF4 and YIPF3 formed numerous punctate structures that exhibited colocalization with lysosomal-associated membrane protein 1 (LAMP1) and LC3B. This process was dependent on FIP200 and vacuolar protein sorting 34 (VPS34), indicating the mobilization of YIPF4 into autolysosomes. Treatment with the E1 ubiquitin-activating enzyme inhibitor TAK243 did not affect starvation-triggered formation of YIPF4 puncta, indicating that ubiquitylation is not involved in YIPF4-mediated Golgiphagy. Proteomic analysis revealed accumulation of 79 Golgi CAPs in *YIPF4*^*–/–*^ cells. Thus, Golgi-resident YIPF3 and YIPF4 proteins fulfil the criteria for receptors mediating Golgiphagy. They interact with ATG8-related autophagy proteins and mediate degradation of a cohort of Golgi membrane proteins. They are themselves engulfed by autophagosomes and delivered to lysosomes for degradation.

**Figure 1:**
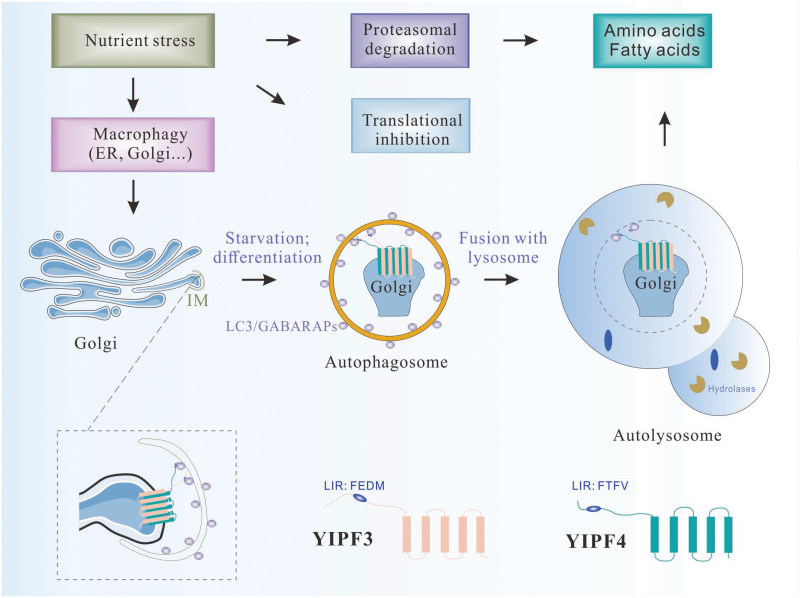
YIPF3 and YIPF4 act as Golgiphagy receptors. Under nutrient stress conditions, the proteasome degradation pathway and the autophagy pathway are activated to provide building blocks for the survival of cells. Upon nutrient stress, membranous organelles are prioritized for autophagic degradation. YIPF3 and YIPF4 are transmembrane proteins that are localized on the Golgi. Upon starvation or neural differentiation, YIPF3 and YIPF4 form heterodimers, and the LIRs located at the N-termini interact with LC3/GABARAPs to mediate the delivery of the Golgi to the lysosome for degradation.

To explore the physiological function of YIPF4, the authors examined its impact on Golgi remodelling during neuronal differentiation. Remarkably, the differentiation of *YIPF4*^*–/–*^ human embryonic stem (ES) cells into neurons resulted in selective accumulation of Golgi membrane proteins to a degree comparable to that in induced *ATG12*^*–/–*^ neurons. These results underscore a broader role of YIPFs in autophagy-mediated Golgi remodelling under nutrient stress and cellular state changes.

CALCOCO1 has been identified as a soluble ERphagy receptor [8]. CALCOCO1 mediates ERphagy by interacting with the ER transmembrane protein VAP (vesicle-associated membrane protein-associated protein) and also with LC3/GABARAP proteins [8]. CALCOCO1, a small fraction of which is localized to the Golgi, also acts as a receptor for Golgiphagy by binding to the Golgi palmitoyl-transferase zinc finger DHHC-type palmitoyltransferase 17 (ZDHHC17 or ZD17) during starvation [8]. Depletion of CALCOCO1 results in the expansion of the Golgi complex and the accumulation of Golgi proteins [8]. Distinct from CALCOCO1 depletion, the Golgi morphology was largely unaffected in cells depleted of YIPF3 and YIPF4. YIPFs and CALCOCO1 appear to employ distinct mechanisms for Golgiphagy. Proteomic analysis showed that *CALCOCO1*^*–/–*^ had no effect on the turnover of YIPF4 and Golgi membrane proteins. Meanwhile, CALCOCO1 turnover in response to nutrient stress did not depend on YIPF3 and YIPF4 [7]. In *Drosophila*, GMAP (Golgi microtubule-associated protein), a *cis*-Golgi protein, also interacts with ATG8a, regulates Golgi turnover, and controls the size and morphology of the Golgi complex [9]. Thus, different species and different tissues under different stress and physiological conditions may employ different Golgiphagy receptors.

This seminal study provides compelling evidence to show that autophagy triggered by nutrient stress exhibits selectivity and prefers to degrade membrane-enclosed organelles. The LIR motif-containing transmembrane proteins YIPF3/4 act as receptors for Golgiphagy during nutrient stress and neuronal differentiation. This study also raises several intriguing questions. What is the mechanism by which YIPF3/4 governs the capture of the Golgi complex? Do autophagosomes engulf whole Golgi cisterns or just portions? If only a portion of a cistern is degraded, what is the mechanism that drives the scission process? If the Golgi function is impaired, such as by accumulation of misfolded secretory proteins, are the same receptors utilized to target the Golgi complex for degradation? Is ERphagy coordinated with Golgiphagy, and if so, how? What is the signal that initiates and regulates Golgiphagy? Addressing these questions will lead to a better understanding of the mechanisms that coordinate selection of cargoes for autophagic degradation to help cells cope with nutrient stress.

## References

[1] Nakatogawa H. Nat Rev Mol Cell Biol 2020;21:439–58. 32372019 10.1038/s41580-020-0241-0

[2] Zhao YG, Codogno P, Zhang H. Nat Rev Mol Cell Biol 2021;22:733–50. 34302147 10.1038/s41580-021-00392-4 PMC8300085

[3] Vargas JNS, Hamasaki M, Kawabata T et al. Nat Rev Mol Cell Biol 2023;24:167–85. 36302887 10.1038/s41580-022-00542-2

[4] Sawa-Makarska J, Abert C, Romanov J et al. Nat Cell Biol 2014;16:425–33. 24705553 10.1038/ncb2935 PMC4009068

[5] Johansen T, Lamark T. J Mol Biol 2020;432:80–103. 31310766 10.1016/j.jmb.2019.07.016

[6] Chino H, Mizushima N. Cold Spring Harb Perspect Biol 2023;15:a041256. 35940904 10.1101/cshperspect.a041256 PMC9808580

[7] Hickey KL, Swarup S, Smith IR et al. Nature 2023;623:167–74. 37757899 10.1038/s41586-023-06657-6 PMC10620096

[8] Nthiga TM, Shrestha BK, Bruun JA et al. J Cell Biol 2021;220:e202006128. 33871553 10.1083/jcb.202006128 PMC8059076

[9] Rahman A, Lorincz P, Gohel R et al. Cell Rep 2022;39:110903. 35649355 10.1016/j.celrep.2022.110903 PMC9637997

